# Restoration of liver sinusoidal cell phenotypes by statins improves portal hypertension and histology in rats with NASH

**DOI:** 10.1038/s41598-019-56366-2

**Published:** 2019-12-27

**Authors:** Miren Bravo, Imma Raurell, Diana Hide, Anabel Fernández-Iglesias, Mar Gil, Aurora Barberá, Maria Teresa Salcedo, Salvador Augustin, Joan Genescà, María Martell

**Affiliations:** 1 Liver Unit, Department of Internal Medicine, Hospital Universitari Vall d’Hebron, Institut de Recerca Vall d’Hebron (VHIR), Universitat Autonoma de Barcelona, Barcelona, Spain; 2 Centro de Investigación Biomédica en Red de Enfermedades Hepáticas y Digestivas (CIBEREHD), Instituto de Salud Carlos III, Madrid, Spain; 3 Liver Vascular Biology Research Group, Hepatic Hemodynamic Lab. IDIBAPS-Hospital Clínic, Barcelona, Spain; 4 Department of Pathology, Hospital Universitari Vall d’Hebron, Barcelona, Spain

**Keywords:** Non-alcoholic steatohepatitis, Portal hypertension

## Abstract

Non-alcoholic steatohepatitis (NASH) is a common chronic liver disorder in developed countries, with the associated clinical complications driven by portal hypertension (PH). PH may precede fibrosis development, probably due to endothelial dysfunction at early stages of the disease. Our aim was to characterize liver sinusoidal endothelial cell (LSEC) dedifferentiation/capillarization and its contribution to PH in NASH, together with assessing statins capability to revert endothelial function improving early NASH stages. Sprague-Dawley rats were fed with high fat glucose-fructose diet (HFGFD), or control diet (CD) for 8 weeks and then treated with simvastatin (sim) (10 mg·kg^−1^·day^−1^), atorvastatin (ato) (10 mg·kg^−1^·day^−1^) or vehicle during 2 weeks. Biochemical, histological and hemodynamic determinations were carried out. Sinusoidal endothelial dysfunction was assessed in individualized sorted LSEC and hepatic stellate cells (HSC) from animal groups and in whole liver samples. HFGFD rats showed full NASH features without fibrosis but with significantly increased portal pressure compared with CD rats (10.47 ± 0.37 mmHg *vs* 8.30 ± 0.22 mmHg; p < 0.001). Moreover, HFGFD rats showed a higher percentage of capillarized (CD32b^−^/CD11b^−^) LSEC (8% *vs* 1%, p = 0.005) showing a contractile phenotype associated to HSC activation. Statin treatments caused a significant portal pressure reduction (sim: 9.29 ± 0.25 mmHg, p < 0.01; ato: 8.85 ± 0.30 mmHg, p < 0.001), NASH histology reversion, along with significant recovery of LSEC differentiation and a regression of HSC activation to a more quiescent phenotype. In an early NASH model without fibrosis with PH, LSEC transition to capillarization and HSC activation are reverted by statin treatment inducing portal pressure decrease and NASH features improvement.

## Introduction

The drastic increase in obesity prevalence due to lifestyle changes is bringing on a chronic liver disease epidemic, non-alcoholic fatty liver disease (NAFLD). NAFLD is considered the hepatic component of the metabolic syndrome covering a broad spectrum of liver damage ranging from simple steatosis, which might progress to nonalcoholic steatohepatitis (NASH), fibrosis, cirrhosis and finally, hepatocellular carcinoma^1^.

Portal hypertension (PH) is responsible for most of the clinical complications associated to chronic liver diseases^2,3^. Several studies have verified the presence of PH in NAFLD even without the presence of fibrosis in patients and animal models, raising the hypothesis that PH precedes the development of cirrhosis in patients with NAFLD^4,5^. Sinusoidal endothelial dysfunction with diminished nitric oxide (NO) production has been identified as a major contributor to the increase in the intrahepatic vascular tone in cirrhosis and thus to the raised portal pressure (PP)^6–8^.

Differentiated liver sinusoidal endothelial cells (LSEC) are characterized by the presence of fenestrae and absence of a basement membrane, allowing a direct communication between sinusoidal blood and the subendothelial space of Disse^9–12^. This highly specialized phenotype includes CD32b surface antigen expression, which is correlated with the presence of fenestrations^13^. Under pathological conditions, LSEC lose this distinctive properties together with a modification of surface markers expression including loss of CD32b^14–16^. This dedifferentiation towards a common vascular endothelial phenotype, known as “capillarization”, precedes liver fibrosis and HSC activation in various liver diseases, including NAFLD^14,17–19^.

Recent studies have shown that statins could improve hepatic steatosis and therefore ameliorate NASH activity^20–22^. Although current evidence on their safety is still a matter of debate, statins have also been considered to exert lipid-independent pleiotropic effects through 3-hydroxy-3-methyl-glutaryl-coenzyme A (HMG-CoA) reductase inhibition, reducing the synthesis of isoprenoids that serve as lipophilic attachments for various signaling proteins, such as the small Ras, Rac, and Rho GTPases. Mechanisms involved in the beneficial effects of statins include improvement of endothelial dysfunction, increasing endothelial nitric oxide synthase (eNOS) activity and inhibition of RhoA/Rho-kinase^23–26^. Since LSEC phenotype maintenance requires autocrine production of NO^12^, statins might be considered for the prevention of LSEC capillarization in NASH.

In the present study, we analyzed individualized subtypes of LSEC by cell sorting, from a diet induced NASH rat model with the aim of demonstrating the contribution of LSEC dedifferentiation/capillarization and hepatic stellate cell (HSC) activation to NASH liver alterations and PH. We also provided information regarding sinusoidal endothelial protective effects exerted by statins capable of improving fatty liver disease pathophysiology.

## Material and Methods

### Experimental design

The study design and assignation of animals for the different experimental analysis is depicted in Supplementary Fig. 1. The results are from single experiments with multiple rats. The n values are for biological replicates.

### Animals and diet

Male Sprague-Dawley OFA rats (Charles River Laboratories, L’Arbresle, France) weighing 200–220 g were used for the previously described NASH model^5^ and housed under 12 h light/dark cycle at constant temperature and humidity. Animals were fed *ad libitum* with a high fat glucose-fructose diet (HFGFD) or control diet (CD). HFGFD consisted of 30% fat (butter, coconut oil, palm oil, beef tallow) with mainly saturated fatty acids (5.73 Kcal/g), supplemented with cholesterol (1 g/Kg) (Ssniff Spezialdiaten GmbH, Soest, Germany), and a beverage of glucose-fructose (42 g/L, 45% glucose-55% fructose). Control diet consisted of a grain based chow that contents 4.8% fat (3.43 Kcal/g) (Safe-150, SAFE, Augy, France) and tap water. Body weight and food consumption was weekly monitored.

All procedures were conducted in accordance with European Union Guidelines for Ethical Care of Experimental Animals (EC Directive 86/609/EEC for animal experiments) and approved (file number: 9481) by the Animal Care Committee of the Vall d’Hebron Institut de Recerca (VHIR, Barcelona, Spain) and conducted in the animal facilities of VHIR.

### Drug administration

Eight-week HFGFD fed rats received daily oral doses of statins or vehicle for 2 weeks. 10 mg/kg/day simvastatin (Ratiopharm, Madrid, Spain) (HFGFD-Sim), 10 mg/kg/day atorvastatin (Almirall, Barcelona, Spain) (HFGFD-Ato) or equivalent volume of water (HFGFD-Veh) were administered by gastric gavage. Animals with control diet also received water intragastrically (CD-Veh). Each group continued to have access to the original diet during the entire treatment period. Experiments were performed ninety minutes after the last dose of statin or vehicle.

Methods followed to perform hemodynamic measurements, biochemical parameters determination, as well as for Western Blot and RT-qPCR are described in detail in the Supplementary Material.

### Histological analyses

Liver samples were fixed in 4% formaldehyde for 24 h, embedded in paraffin and sectioned in 4 μm thick slides. Hematoxylin-eosin was used to assess liver parenchyma and picrosirius red to analyze fibrosis. Samples were evaluated twice, with an interval of two years, by an expert liver pathologist blinded to animal interventions.

NAFLD activity score (NAS), obtained from the grading of steatosis, hepatocellular ballooning and lobular inflammation as the NASH-CRN criteria (27) (see Supplementary Table 1 for more detail). The diagnosis of NASH was made as per current standards, based on the concurrence of steatosis, ballooning and inflammation and a NAS ≥3. Fibrosis was scored according to the NASH-CRN system, ranging from F0 (no fibrosis) to F4 (cirrhosis).

### LSEC and HSC isolation

LSEC and hepatic stellate cells (HSC) were isolated from CD-Veh (*n* = 4 for LSEC; n = 5 for HSC), HFGFD-Veh (*n* = 4 for LSEC; n = 5 for HSC), HFGFD-Sim (*n* = 4 for LSEC; n = 5 for HSC) and HFGFD-Ato (n = 4 for LSEC; n = 5 for HSC) rat livers as previously described^24^. Briefly, livers were perfused with collagenase (also with pronase and DNase for HSC), excised and digested. Resulting cells were filtered and centrifuged to eliminate hepatocytes. For LSEC the supernatant was centrifuged in a two phase Percoll gradient (25%/50%). The central fraction containing LSEC and Kupffer cells (KC) was collected, and seeded in a non-coated plate for 30 min. Non-adherent LSEC were seeded in collagen-coated culture plates, incubated for 45 min and washed afterwards. For HSC the supernatant was centrifuged in Optiprep gradient (11.5%) and the fraction containing HSC was collected, seeded in a non-coated plate overnight and washed afterwards (See Supplementary Material for a more detailed explanation).

### Fluorescence-activated cell sorting

LSEC were harvested and 3 × 10^6^ cells washed with PBS 5% FBS. Cells were then incubated with fluorescent labelled antibodies against CD32b-FITC (1:10; Novus Biologicals, Littleton, CO, USA) and CD11b/c-APC (1:10; Miltenyi Biotec, Bergisch Gladbach, Germany) for 1 h, and resuspended in 500 µL of PBS 5% FBS. Two-color flow cytometry was performed on a FACS-Aria flow cytometer (BD Biosciences, San Jose, CA, USA). Voltages were based on unstained cells and compensation was set using single-stained positive controls for each color. Viable cells were sorted by DAPI staining, doublets and aggregates were excluded from analysis based on the forward scatter (FSC)-H and FSC-A profile and KC or macrophages were excluded by gating CD11b/c cells. Cells were collected in 1 ml Trizol (Thermo Fisher scientific, Waltham, MA, USA) for RNA extraction. Results were evaluated with FCS Express 4 Flow Research Edition.

### HSC vitamin A detection

HSCs were cultured on well chamber slides (Ibidi, Martinsried, Germany) and fixed with 4% paraformaldehyde for 10 min. Vitamin A fluorescence was detected by using a 350/50 excitation filter and a 450/50 nm emission filter for detection in a Leica DM microscope. Then cells were rinsed with dH_2_O and incubated with 100% propyleneglycol for 5 min. Cells were stained with 0.5% Oil Red O (Sigma Aldrich, Darmstadt, Germany) in 100% propyleneglycol for 15 min. After washing with 85% propyleneglycol and dH_2_O, cells were allowed to dry. Accumulation of lipid droplets in HSCs was quantified by Fiji software.

### Statistical analysis

Statistical analysis was performed with the Sigmastat 3.0 package (San Jose, CA, USA). All results are expressed as mean ± standard error of the mean (SEM) and compared by Student’s t, Mann-Whitney or one-way ANOVA, followed by a post-hoc test when adequate. Differences were considered significant at a p value < 0.05.

## Results

### Statins effect on bodyweight gain and biochemical parameters

HFGFD fed animals showed a 20% of weight gain difference compared to CD rats. Treatment with statins (simvastatin or atorvastatin) had no influence on bodyweight compared with HFGFD rats treated with vehicle (Supplementary Fig. 2A).

Changes in fasting blood glucose and insulin levels induced by HFGFD (Supplementary Table 2), indicating insulin resistance in these animals, were further reflected in a significant increase in the HOMA-IR index (Supplementary Fig. 2B). In statin treated animals, the levels of these parameters reached values comparable to those of CD-Veh group, suggesting an improvement of insulin sensitivity associated with statin treatment. ALT, triglyceride and albumin values were also changed after HFGFD. ALT levels were significantly reduced with atorvastatin treatment, while triglycerides reduction was assessed by both statins.

Statin treatments had only mild muscular toxicity in 7.7% of HFGFD-Sim rats and in 12.5% of HFGFD-Ato rats. Hepatic toxicity was absent in all animals (Supplementary Table 3).

### Statins ameliorate histological NASH induced by HFGFD

**Figure 1:**
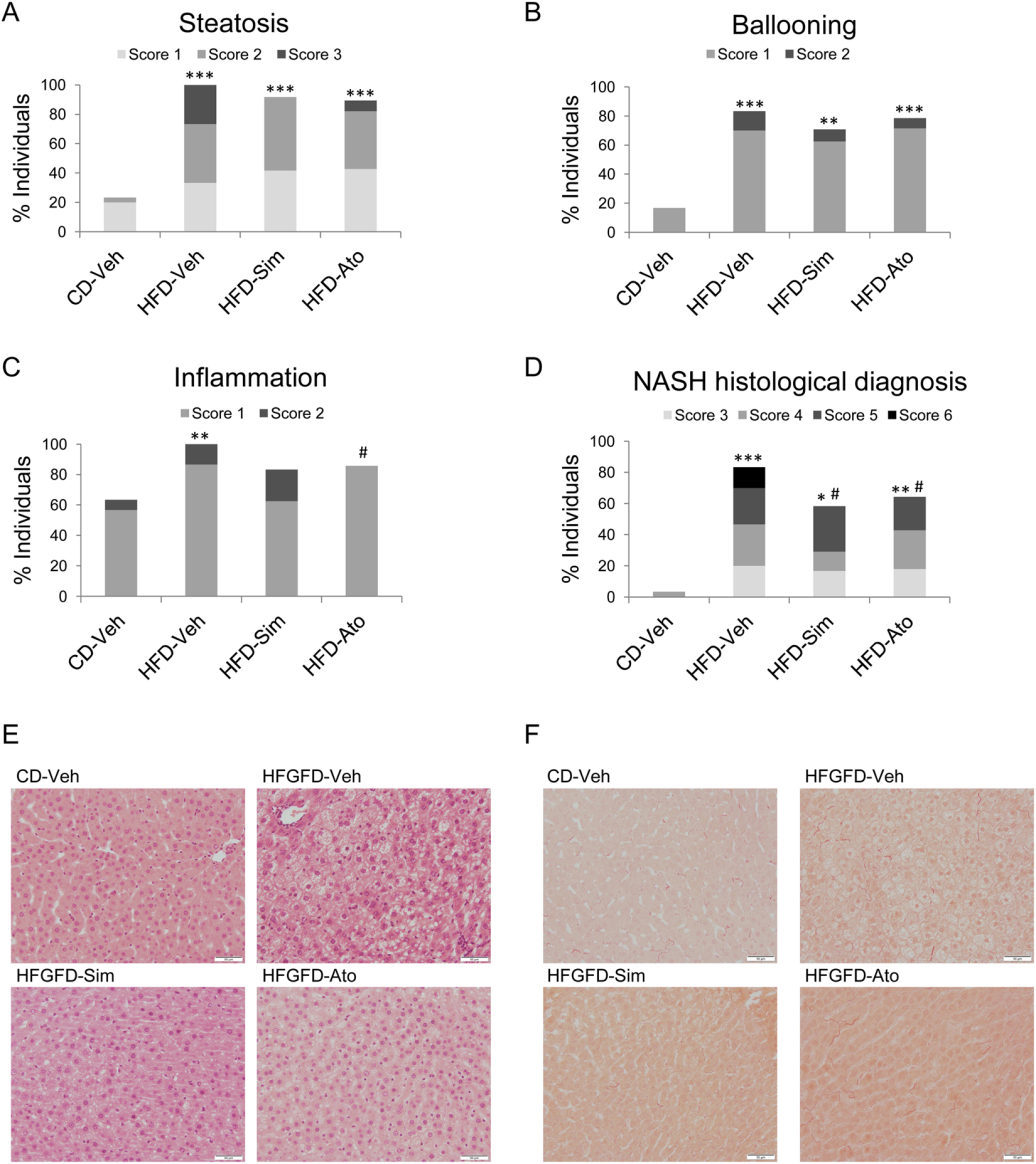
Liver histology. Histological evaluation of NASH following the NASH-CRN Histological Scoring in control diet (CD-Veh, n = 15), and high-fat glucose-fructose diet rats treated with vehicle (HFGFD-Veh, n = 15), simvastatin (HFGFD-Sim, n = 12) or atorvastatin (HFGFD-Ato, n = 14). Bar diagrams representing the average percentage of two separated histological analysis of individuals that present (**A**) steatosis, (**B**) hepatocelular ballooning and (**C**) lobular inflammation, and (**D**) histological diagnosed NASH (defined as NAFLD activity score ≥3 and concurrence of steatosis, hepatocellular ballooning and lobular inflammation). Increasing scores are depicted by light, medium and dark grey, and black. Representative images (20x magnification) of liver sections stained with (**E**) Hematoxylin-Eosin and (**F**) Sirius Red to asses fibrosis. Mann Whitney U test was used to compare the percentages of each score by pairs of groups for each item (*p < 0.05; **p < 0.01; ***p < 0.001 compared with CD-Veh; ^#^p < 0.05 compared to HFGFD-Veh).

HFGFD-Veh animals revealed significantly higher percentages of steatosis, hepatocelular ballooning and lobular inflammation compared to CD-Veh (p < 0.001; p < 0.001; and p = 0.005, respectively) (Fig. 1A–C,E). HFGFD fed rats developed histological NASH in a higher proportion compared to CD-Veh rats (p < 0.001) (Fig. 1D). Steatosis, ballooning and inflammation scores were lower in the case of statin treated animals, resulting in a decreased percentage of individuals with histological NASH compared to HFGFD-Veh (HFGFD-Sim p = 0.03; HFGFD-Ato p = 0.04), together with lower NAS activity scores (Fig. 1A–E).

Sirius Red stained liver sections from ten-week CD and HFGFD rats treated with vehicle, simvastatin or atorvastatin are shown in Fig. 1F. No animal of any group developed fibrosis.

### Statin treatments ameliorate hepatic hemodynamics and endothelial dysfunction in liver microvasculature

Ten-week HFGFD induced a significant PP increase compared with CD (26% increase, p < 0.001) (Table 1). In this animal model of NASH, both simvastatin and atorvastatin treatment significantly reduced PP levels compared to vehicle administration (11.24% reduction, p = 0.006, and 13.85% reduction, p < 0.001, for HFGFD-Sim and HFGFD-Ato, respectively) without changing systemic hemodynamics. PP reduction in both treatments is paralleled to a non-significant decrease in IHVR in both groups compared to HFGFD-Veh.

**Table 1:** Hemodynamic measurements.

	CD	HFGFD	HFGFD	HFGFD
	Vehicle	Vehicle	Simvastatin	Atorvastatin
MAP (mmHg)	108.85 ± 4.86	109.88 ± 3.84	105.29 ± 7.26	108.76 ± 5.90
SMABF (mL/[min·100 g])	3.86 ± 0.58	3.36 ± 0.49	3.10 ± 0.46	3.34 ± 0.37
SMAR (mmHg/mL·min·100 g)	31.98 ± 4.27	36.46 ± 4.79	35.52 ± 2.92	36.37 ± 3.11
PP (mmHg)	8.30 ± 0.22***	10.47 ± 0.37	9.29 ± 0.25**	8.85 ± 0.30***
PBF (mL/[min·100 g])	2.56 ± 0.15	2.89 ± 0.22	3.00 ± 0.35	2.81 ± 0.24
IHVR (mmHg/mL·min·100 g)	3.38 ± 0.27	3.62 ± 0.34	3.42 ± 0.46	3.17 ± 0.31
Heart rate (BPM)	328.03 ± 7.14	322.71 ± 10.09	325.06 ± 8.60	324.51 ± 9.30

Values are expressed as mean ± SEM in CD-Vehicle (n = 15), HFGFD-Vehicle (n = 15), HFGFD-Simvastatin (n = 12) and HFGFD-Atorvastatin (n = 14). MAP, mean arterial pressure; SMABF, superior mesenteric artery blood flow; SMAR, superior mesenteric artery resistance; PP, portal pressure; PBF, portal blood flow; IHVR, intrahepatic vascular resistance; BPM, beats per minute. *p < 0.05, **p < 0.01, ***p < 0.001 compared to HFGFD-vehicle (ANOVA).

Whole liver protein expression levels of KLF2 by Western blot showed that HFGFD-Veh rats presented a reduction in the hepatic levels of KLF2, compared to CD-Veh rats, being partially recovered in the groups treated with statins (Supplementary Fig. 3). We also assessed protein kinase B (AKT) and eNOS phosphorylation in liver tissue samples from all groups. HFGFD-Veh rats showed significantly reduced levels of P-Akt and P-eNOS compared to CD-Veh rats (Supplementary Fig. 3). After treatment with atorvastatin, this situation tended to revert, showing increased protein expression levels of P-eNOS and P-AKT in comparison to HFGFD-Veh animals. However, although P-eNOS expression levels recovered in HFGFD-Sim rats, P-AKT levels were only partially increased.

### Statins prevent HFGFD induced LSEC capillarization

**Figure 2:**
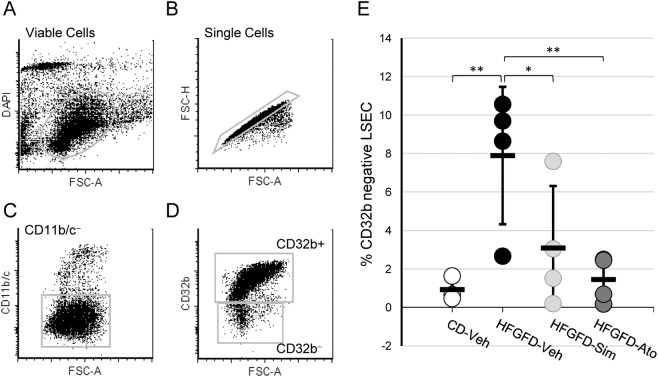
Sorting of LSEC subtypes by flow cytometry. Gating strategy for analyses and sorting of primary isolated LSEC. (**A**) Viable cells within the analyzed sample. (**B**) Single cells. Aggregates and doublets are excluded (**C**) CD11b/c-positive Kuppffer cells are excluded. (**D**) LSEC phenotypes: CD32b^+^ (specialized/differentiated) and CD32b^−^ (capillarized/dedifferentiated). (**E**) Percentage of CD32b^**−**^ cells indentified in control diet (CD-Veh, n = 4) and HFGFD rats after vehicle (HFGFD-Veh, n = 4), simvastatin (HFGFD-Sim, n = 4) or atorvastatin (HFGFD-Ato, n = 4) treatment. Group values are expressed as mean ± SEM. Percentage of each individual is shown as a dot. *p < 0.05; **p < 0.01 compared to HFGFD-vehicle (ANOVA).

After sorting viable cells and excluding cell aggregates and KCs (Fig. 2A–C), primary LSEC were subtyped as differentiated CD32b^+^/CD11b/c^**−**^ cells or dedifferentiated CD32b^−^/CD11b/c^−^ LSEC (Fig. 2D).

To rule out the presence of other liver main cells in the dedifferentiated subpopulation, we quantified the expression of specific genes for each cellular type: desmin for HSC, F4/80 for Kupffer cells and Cyp3A2 for hepatocytes. Supplementary Fig. 4 demonstrates the complete absence of expression of any of these genes in dedifferentiated LSEC from the different groups of animals.

Although in a very small proportion (1%), control animals also displayed CD32b^−^/CD11b/c^−^ LSEC. By contrast, we found a significant increase in the proportion of CD32b^−^/CD11b/c^−^ LSEC in ten-week HFGFD fed rats compared with those fed with control diet (8%, p = 0.005) (Fig. 2E). Moreover, treatment with both statins significantly decreased the percentage of these potentially dedifferentiated cells compared to vehicle animals from 8% to 3% (p = 0.024) and 1.5% (p = 0.005), for simvastatin and atorvastatin, respectively (Fig. 2E).

### LSEC subtypes are different between CD and HFGFD rats

**Figure 3:**
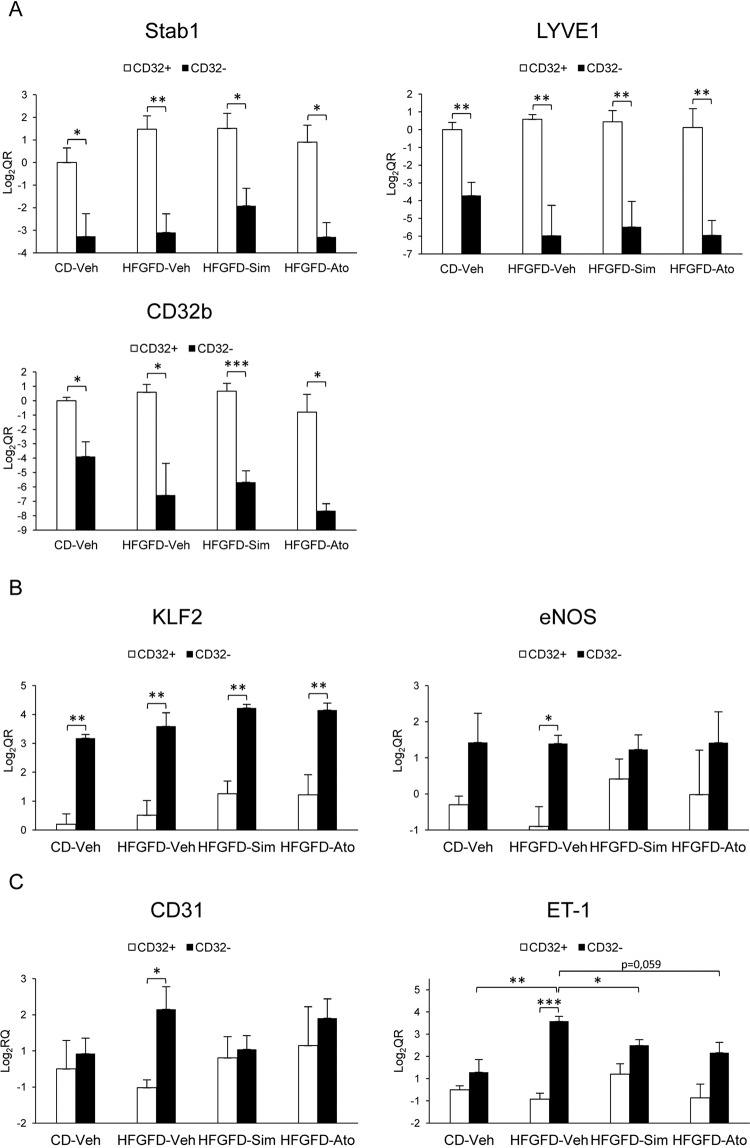
Gene expression analysis of LSEC subtypes. Relative quantitation of mRNA expression of (**A**) Stab1, LYVE-1 and CD32b; (**B**) KLF2 and eNOS; and (**C**) CD31 and ET-1 by qRT-PCR, expressed as fold change LOG2 ratio in CD32b+ and CD32b- LSEC isolated from (CD-Veh, n = 4) and HFGFD rats after vehicle (HFGFD-Veh, n = 4), simvastatin (HFGFD-Sim, n = 4) or atorvastatin (HFGFD-Ato, n = 4) treatment. GAPDH was used as endogenous control and results were normalized to CD32b^+^ cells from CD-Veh group. mRNA levels are expressed as mean ± SEM. *p < 0.05, **p < 0.01; ***p > 0.001(unpaired t test).

We then characterized CD32b^−^ LSEC from control and HFGFD animals based on their genetic expression profile (Fig. 3). We first analyzed the expression level of three genes related to endocytosis, STAB1, LYVE-1 and CD32b (Fig. 3A), the most distinctive feature of specialized LSEC. CD32b^**−**^ cells had significantly lower mRNA levels of STAB1, LYVE-1 and CD32b in contrast to CD32b^+^ cells in all groups of animals.

We also examined responsive genes to fluid shear stress, such as KLF2 and eNOS (Fig. 3B), in order to analyze the microvascular regulation capacity of these cells. KLF2 mRNA levels in CD32b^−^ cells were significantly higher compared to CD32b^+^ cells both in CD-Veh and HFGFD-Veh rats. eNOS mRNA levels were also higher in CD32b^−^ cells, but reaching significance only in the HFGFD-Veh group, probably due to simultaneous decreased expression in CD32b^+^ cells.

Finally, we analyzed the gene expression of CD31, associated with LSEC dedifferentiation, and ET-1, related to vasoconstriction (Fig. 3C). There were no differences regarding to these endothelial dysfunction related genes between CD32b^+^ and CD32b^−^ in CD-Veh rats. However, when analyzing CD32b^+^ vs. CD32b^−^ cells from HFGFD-Veh animals, a significant increase in both CD31 and ET-1 mRNA levels in CD32b^−^ cells was observed.

### Statins restore the genetic expression profile of capillarized LSEC

Statins did not produce significant differences in the expression of endocytosis related genes, STAB1, LYVE-1 and CD32b, in CD32b^+^ cells, nor in the CD32b^−^ cells of NASH animals (Fig. 3A). However, after treatment, there was a trend to increase KLF2 levels in both cellular subtypes, surpassing the expression levels achieved in HFGFD-Veh (Fig. 3B). This rise in KLF2 is translated into a non-significant increment in eNOS mRNA levels in CD32b^+^ cells, whereas in CD32b^−^ cells eNOS remains unchanged (Fig. 3B). In addition, neither simvastatin nor atorvastatin treatment caused any difference in the gene expression of CD31 or ET-1 in CD32^+^ cells of NASH animals (Fig. 3C). However, in CD32b^**−**^ both simvastatin and atorvastatin treated rats expressed significantly lower levels of ET-1 (sim: p = 0.03; ato: p = 0.059), suggesting a reversion of the CD32b− vasoconstrictive phenotype by these drugs (Fig. 3C). Expression levels of CD31 gene in CD32b− cells followed the same pattern, with an increase in HFGFD-Veh and a decrease of these levels due to statins, mostly in HFGFD-Sim, although without statistical significance (Fig. 3C).

### Statins restore HSC quiescent phenotype preventing ET-1 induced activation

**Figure 4:**
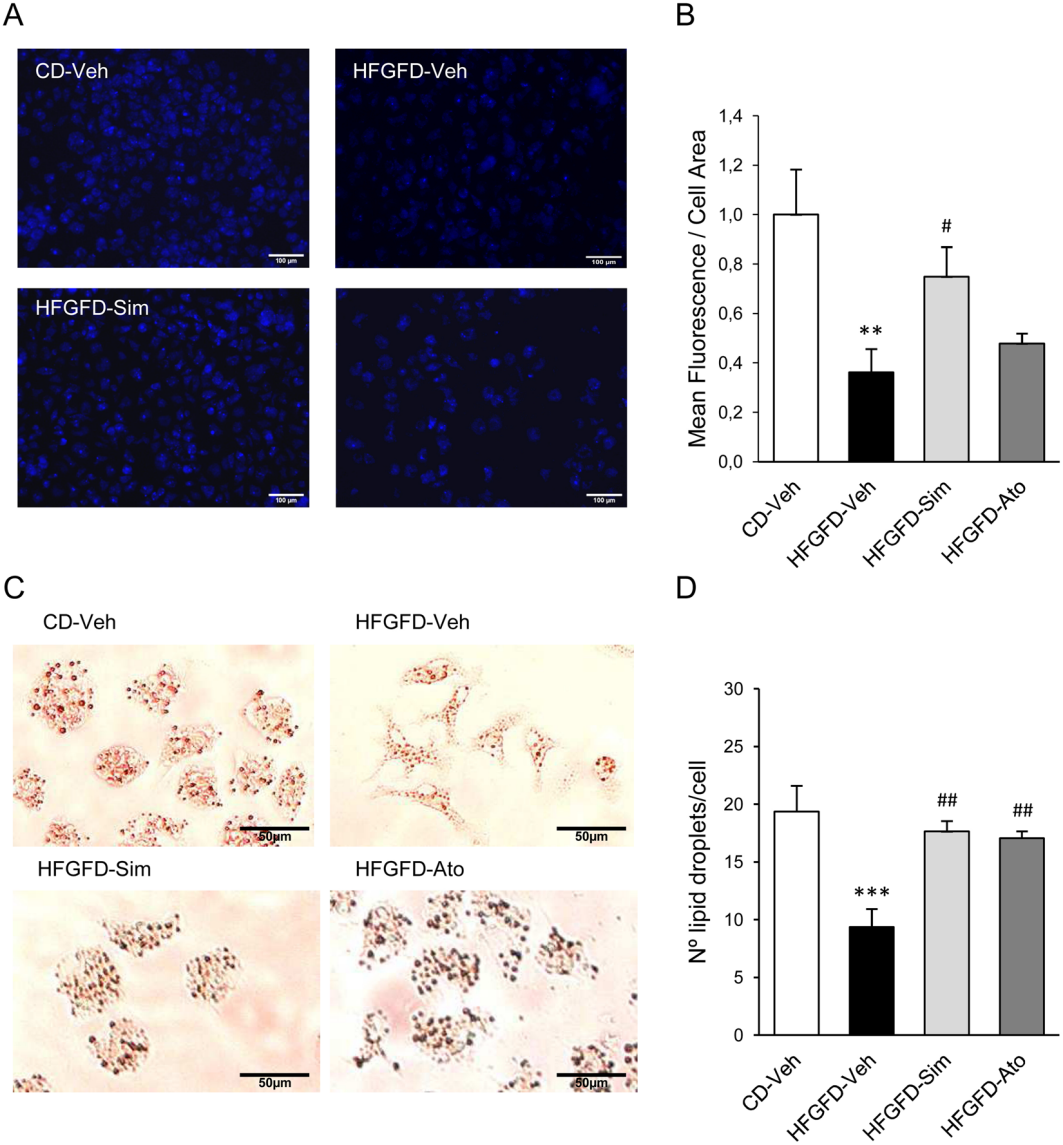
Detection of vitamin A in HSC. Stored Vitamin A quatitation in primary isolated HSCs from control diet (CD-Veh, n = 5) and HFGFD rats after vehicle (HFGFD-Veh, n = 5), simvastatin (HFGFD-Sim, n = 5) or atorvastatin (HFGFD-Ato, n = 5) treatment. (**A**) Representative images of freshly isolated HSCs visualized using vitamin A autofluorescence. (**B**) Quantification of mean autofluorescence/cell area. (**C**) Representative images of Oil Red O staining of HSCs. (**D**) Number of lipid droplets/cell. Values are expressed as mean ± SEM; **p < 0.01, ***p < 0,001 compared to CD-Veh; ^#^p < 0.05, ^##^p < 0.01 compared to HFGFD-Veh (ANOVA).

In order to characterize HSC activation level, we analyzed the amount of vitamin A by autofluorescence and Oil Red O staining. HSC from HFGFD-Veh animals showed a significantly lower mean auto-fluorescence (p = 0.004) (Fig. 4A,B), an elongated spindle morphology (Fig. 4C) and a significantly lower number of lipid droplets per cell (p < 0.001) **(**Fig. 4D) compared with HSC from CD-Veh. HSC from simvastatin treated rats had a significantly higher mean auto-fluorescence (p = 0.03) compared with HSC from HFGFD-Veh, while cells obtained from the atorvastatin treated group showed a non-significant increasing trend (Fig. 4B). Moreover, Oil Red O staining revealed a more quiescent appearance with a star-like morphology in HSC from animals treated with both statins (Fig. 4C), together with a significant higher number of lipid droplets compared to HFGFD-Veh (p = 0.005 and p = 0.009, for simvastatin and atorvastatin, respectively) (Fig. 4D).

**Figure 5:**
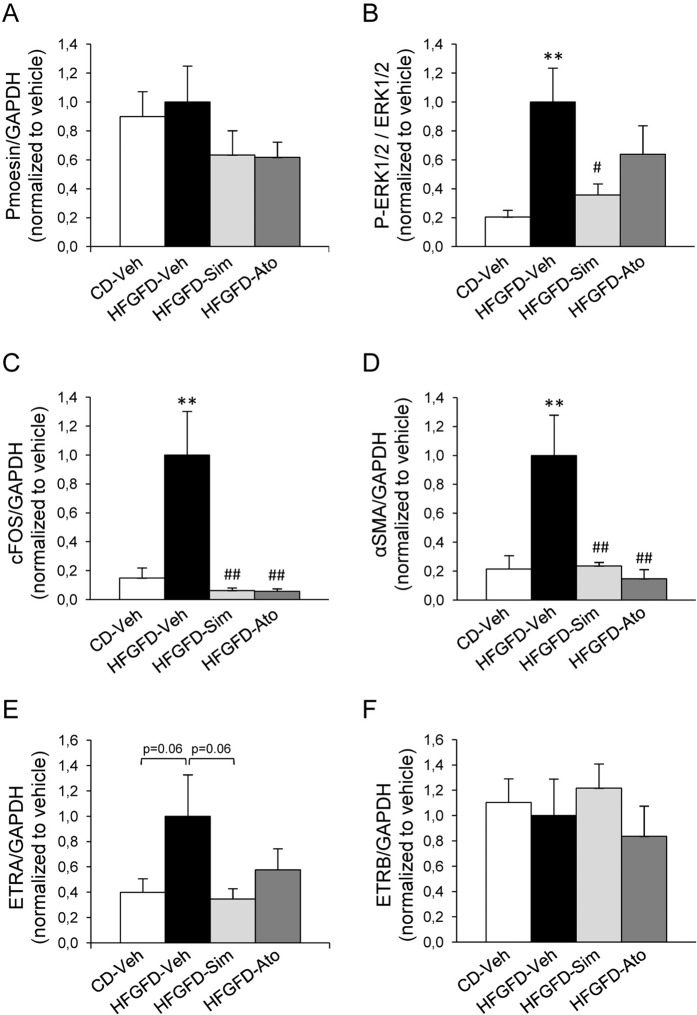
Endothelin pathway markers in HSC. Bar diagrams show protein quantification of (**A**) P-moesin, (**B**) P-ERK1/2 (**C**) cFOS (**D**) αSMA (**E**) ETRA and (**F**) ETRB in HSC from control diet (CD-Veh, n = 5), and high-fat glucose-fructose diet rats treated with vehicle (HFGFD-Veh, n = 5), simvastatin (HFGFD-Sim, n = 5) or atorvastatin (HFGFD-Ato, n = 5). Full-length gels are included in Supplementary Fig. 5. GAPDH was used as loading control. Protein levels are normalized to HFGFD-Veh group and expressed as mean ± SEM. **p < 0.01 compared to CD-Veh; ^#^p < 0.05, ^##^p < 0.01 compared to HFGFD-Veh (ANOVA).

Finally, we evaluated ET-1 signaling pathways by Western blot analysis in isolated hepatic stellate cells (Fig. 5 and Supplementary Fig. 5). There were no differences in moesin phosphorylation levels in HSC from HFGFD-Veh compared to those from CD-Veh (Fig. 5A). However, they exhibited a significant increase in P-ERK1/2, cFOS and αSMA (Fig. 5B–D). Simvastatin treatment significantly reduced P-ERK1/2 levels, while atorvastatin showed a discrete decrease (Fig. 5B). However, both statins reduced cFOS and αSMA expression levels to those of control diet fed animals (Fig. 5C,D). HFGFD-Veh cells showed an almost significant increase in ETRA, which was avoided by both statin treatments (Fig. 5E). There were no differences in ETRB expression levels between groups (Fig. 5F).

## Discussion

In the present study, we used a diet-induced NASH rat model previously set up in our lab^5^, which develops obesity, insulin resistance, endothelial dysfunction and PH in absence of fibrosis. Here, we demonstrated that statins exert a general improvement in NASH pathophysiology, including histopathology, PP, endothelial dysfunction, and LSEC and HSC phenotypes. In contrast to previous experiences in preclinical models of liver cirrhosis where statins were toxic^25^, the beneficial effects of statins in our model of diet-induced NASH were observed with minimal toxic effects; hepatic toxicity was absent and just few animals had mild muscular toxicity.

In addition to improving the histopathology, showing a reduction of steatosis, ballooning and inflammation, both simvastatin and atorvastatin were able to reduce serum triglycerides and insulin resistance, and atorvastatin also reduced ALT levels. Moreover, they also showed a significant decrease in PP without changes in systemic hemodynamics.

Liver sinusoidal endothelial dysfunction in NAFLD contributes to increased intrahepatic vascular resistance and, therefore, to PH, without structural changes^8,27^. In this sense, PH and endothelial dysfunction can be present at very early stages of NASH, even before the development of fibrosis or inflammation^4^. Our early stage NASH model showed increased PP and decreased eNOS and AKT protein phosphorylation levels, suggesting impaired endothelial dependent vasodilatation. In this study, statins have been shown to be effective in reducing PP, at least in part probably due to insulin resistance improvement and restoration of eNOS activity^23,25,28^. This NO availability increased by statins could also explain the histopathologic improvement shown by our treated HFGFD rats, since both steatosis and inflammation have been related to endothelial dysfunction^29,30^.

We then decided to go further and explore the specific role of LSEC in the intrahepatic resistance regulation. Recent publications described how two distinct subsets of LSEC are arranged following the acinar pattern, located differentially to fulfill distinct physiological functions under normal conditions: pericentral zone LSEC, with great expression of CD32b, display highly enriched immune pathways including phagocytosis, while periportal zone LSEC, with little expression of CD32b, have enriched pathways in vessel development^31,32^. We have also found these two subtypes of LSEC in healthy animals; although in a small percentage, we obtained CD32b^−^ cells, most probably corresponding to LSEC in periportal zone. In addition, analysis of gene expression nicely reproduced data from previous studies with LSEC subtypes differentiated as cells with either high or mid-low expression of CD32b, STAB1 and LYVE-1.

Concerning HFGFD fed animals, we found that the percentage of CD32b^−^ LSEC was significantly higher than in healthy rats. The conversion of a healthy LSEC population with almost absence of CD32b^−^ cells, to a significant eightfold increase, might reflect the initial changes in an early stage of NASH. Thus, it seems that a significant part of LSEC lost their specific surface marker in HFGFD rat livers, having a lower proportion of cells expressing CD32b and scavenger receptors, such as LYVE-1 and STAB1. This might turn out in a decreased hepatic blood clearance ability in these animals. Furthermore, when analyzing CD32b^−^ cells from HFGFD-Veh, they expressed higher levels of genes related to capillarization such as CD31 and ET-1. Hence, these cells are not equivalent to those periportal CD32b^−^ cells isolated from CD-Veh animals, but they rather seem to be dedifferentiated LSEC, in a process of capillarization induced by the ten-week HFGFD.

As we mentioned above, statins have been shown to confer vasoprotection to the liver sinusoidal endothelium^23,24,33^. Our experiments suggest that part of this vasoprotection is in maintaining the LSEC differentiated phenotype, since statin treated animals had a significantly lower percentage of CD32b^−^ LSEC, than those treated with vehicle. Experimental models of cirrhosis have shown that, *in vitro*, beneficial effects of statins are directly exerted on LSEC through an overexpression of the transcription KLF2 induced by statins. Here, we have observed that CD32b^+^ LSEC from rats treated with statins showed only discrete increases in mRNA levels of KLF2 and eNOS. In contrast, although CD32b^−^ LSEC showed a similar small increase in KLF2 mRNA levels, the expression of eNOS did not change after treatments, suggesting a blockage in the vasodilatation signaling pathway in capillarized cells. On the other hand, statin treated animals showed significantly lower mRNA expression levels of the vasoconstrictor ET-1 in CD32b^−^ LSEC. It is then worth noting that the beneficial effect of statins reverting the ET-1 expression occurs in CD32b^−^ LSEC where they cause significant changes, but not in CD32^+^ cells. In summary, statins diminish the ratio of dedifferentiated CD32b^−^ LSEC, reversing these cells to their original phenotype that expresses CD32b, Stab1 and Lyve1 at the same levels as in healthy animals. Statins also impede ET-1 overexpression in the remaining CD32b^−^ cells, showing the same features as CD32b^−^ LSEC present in healthy animals, with low expression of Lyve1 and Stab1 and normal expression of ET-1 and CD31.

In addition to changes in LSEC, we have shown that HSCs from HFGFD-Veh rats present the typical myofibroblast-like morphology with loss of vitamin A, featured by the activated phenotype of HSC^34,35^. Also, our observation of an increase in ETRA expression in HSC induced by hypercaloric diet, but not in ETRB, is in accordance with previous results describing that HSC are mainly expressing ETRA when they are in an early phase of activation^36^. Our results showed that treatment with statins not only reduced ETRA protein levels, but also prevented the downstream phosphorylation RAF/MEK/ERK cascade caused by ET-1, which was clearly activated in our model of NASH.

ET-1 has been described to exert a specific sinusoidal constriction mediated by HSC^37^. HSC from HFGFD-Veh rats did not show an increase of Rho-kinase activity, assessed by moesin phosphorylation (the endogenous Rho-kinase substrate), so the contraction is probably due to the increase of intracellular Ca^2+^ caused by endothelin receptors stimulation^38,39^. Likewise, HSC from HFGFD-Veh group expressed significantly increased protein levels of αSMA, which is a well-known stellate cell activation marker, related to a direct action of ET-1 from capillarized LSEC^40^. Therefore, activated HSC from HFGFD-Veh rats are also probably contributing to increased sinusoidal resistance, given their predisposition to contract, thus increasing the intrahepatic pressure. Statins again drastically reverted this increased expression of αSMA.

**Figure 6:**
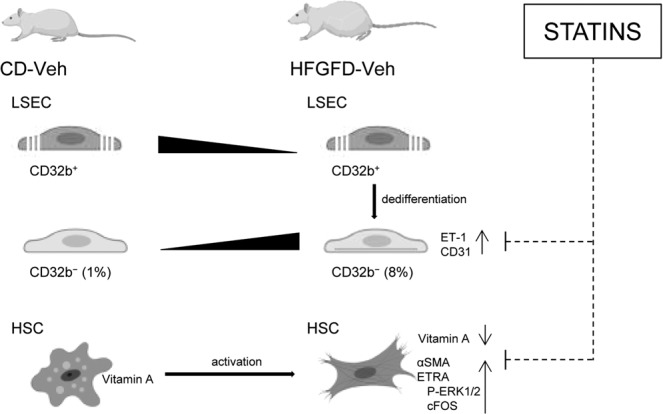
Statins effect on LSEC subtypes and HSC. Control diet animals presented CD32b^+^ and CD32b^**−**^ LSEC subtypes. HFGFD-Veh rats showed increased dedifferentiated CD32b^**−**^ LSEC, expressing higher levels of ET-1 and CD31, and HSC with increased expression of αSMA, ETRA and its downstream signaling proteins, P-ERK1/2 and cFOS, in accordance with an early activation phase. Either directly or through intermediate steps (dashed line), the main beneficial effect of statins is on CD32b^−^ LSEC, decreasing its percentage and preventing ET-1 and CD31 over-expression. HSCs from statin treated animals recovered a more quiescent appearance with vitamin A, reduced aSMA and ETRA levels, likewise its downstream ERK1/2 phosphorylation and cFOS expression (dashed line indicates possible intermediate steps).

Previous studies have shown that LSEC can undergo dedifferentiation in NASH models^17,41^. In the present work, we analyzed for the first time this pathological process in our early stage NASH model, sorting and characterizing this unique “pathological” cell type. We found some of the potential mechanisms by which statins improve intrahepatic microcirculation, especially reducing the number of capillarized LSEC, decreasing ET-1 expression and restoring the normal HSC phenotype (Fig. 6). We could speculate that the LSEC phenotype change in NASH with increased ET-1 expression is responsible for HSC activation and both alterations contribute to PH. Statins by reverting altered LSEC phenotype to a healthy one would deactivate HSC and consequently, PP would decrease. Although we hypothesize that statins act directly in LSEC and that the effect on HSC might be in part a direct consequence of LSEC phenotype improvement, a direct effect of statins in HSC and additional effects of these drugs in other liver cell types cannot be rule out and were not explored here; additional studies are needed to determine these complex interactions. In summary, the results of our study showed that both statins improve NASH histology and PH, at the same time that recover sinusoidal endothelial function by restoring a healthy LSEC and HSC phenotype, leading to PP decrease and consequently, diseases prognosis improvement.

## Supplementary information

Supplementary Information
