# Supplementary material for: Restoration of liver sinusoidal cell phenotypes by statins improves portal hypertension and histology in rats with NASH
Source: Sci Rep. 2019 Dec 27;9:20183. doi: 10.1038/s41598-019-56366-2 (PMC6934751; doi:10.1038/s41598-019-56366-2)

RESTORATION OF LIVER SINUSOIDAL CELL PHENOTYPES BY STATINS IMPROVES PORTAL HYPERTENSION AND HISTOLOGY IN RATS WITH NASH

Author Names:

Miren Bravo[1,2], Imma Raurell[1], Diana Hide[1], Anabel Fernández-Iglesias[2,3], Mar Gil[1], Aurora Barberá[1], Maria Teresa Salcedo[4], Salvador Augustin[1,2], Joan Genesca[1,2], María Martell[1,2]

Affiliations:

1. Liver Unit, Department of Internal Medicine, Hospital Universitari Vall d'Hebron, Institut de Recerca Vall d'Hebron (VHIR), Universitat Autonoma de Barcelona, Barcelona, Spain

2. Centro de Investigacion Biomedica en Red de Enfermedades Hepaticas y Digestivas (CIBEREHD), Instituto de Salud Carlos III, Madrid, Spain

3. Liver Vascular Biology Research Group, Hepatic Hemodynamic Lab. IDIBAPS-Hospital Clínic, Barcelona, Spain

4. Department of Pathology, Hospital Universitari Vall d'Hebron, Barcelona, Spain.

SUPPLEMENTARY INFORMATION

Hemodynamic measurements

Ninety minutes after statin or vehicle administration, 16-hour fasted rats were intraperitoneally anaesthetized with ketamine hydrochloride (100 mg/kg) plus midazolam (5 mg/kg) and body temperature maintained at 37ºC for continuous recording of hemodynamic parameters.

Mean arterial pressure (MAP, mmHg) was measured by catheterization (polyethylene PE-catheter, PE50) of the femoral artery and portal pressure (PP, mmHg) assessed by ileocolic vein catheterization using highly sensitive pressure transducers (Harvard apparatus, Holliston, MA, USA).

Superior mesenteric artery (SMA) blood flow (SMABF, mL/[min*100g]) and portal blood flow (PBF, mL/[min*100g]) were measured with a perivascular ultrasonic transit-time flow probe (1mm diameter, Transonic systems Inc, Ithaca, NY, USA). SMA resistance (SMAR, mmHg/mL*min*100g) and intrahepatic vascular resistance (IHVR, mmHg/mL*min*100g) were calculated as ([MAP-PP]/SMABF) and (PP/PBF), respectively.

Biochemical parameters

Blood samples were collected from the cava vein. Glucose, creatinine, bilirubin, aspartate aminotransferase (AST), alanine aminotransferase (ALT), alkaline phosphatase (ALP), creatinine kinase (CK), total cholesterol, high density lipoprotein (HDL), low density lipoprotein (LDL), triglycerides and albumin were measured with standard methods at the Hospital Vall d'Hebron CORE lab. Insulin was measured using an Elisa kit (EMD Millipore, Billerica, MA, USA). Insulin resistance was estimated applying the homeostasis model of insulin resistance index (HOMA-IR): (fasting insulin (ng/ml)*Fasting glucose (mg/dl))/405.

Hepatic and muscular toxicity due to statin treatment was defined based on ALT and CK levels in vehicle rats. Thus, we defined hepatic toxicity as an increment in ALT levels superior to 150 IU/L and muscular toxicity as an increment in CK levels superior to 8500 IU/L.

LSEC isolation

LSEC were isolated from CD-Veh (*n*=4), HFGFD-Veh (n=4), HFGFD-Sim (n=4) and HFGFD-Ato (n=4) rat livers. Briefly, livers were perfused with collagenase through the portal vein for 10 min at a flow rate of 20 mL/min at 37ºC with Hanks' Balanced Salt Solution (HBSS) without calcium and magnesium containing 12 mM Hepes (pH 7.4), 0.6 mM EGTA and 1.6% bovine serum albumin (BSA). Then, 30 min at a flow rate of 5 mL/min at 37ºC with 0.01% collagenase A, HBSS containing 12 mM Hepes (pH 7.4) and 4 mM CaCl2, excised and in vitro digested for 10 min at 37°C, also with the same buffer. Resulting cells were filtered through a 100 µm nylon filter, collected in cold Krebs buffer and centrifuged at 50 g for 5 min in order to eliminate hepatocytes. The supernatant was then centrifuged at 800 g for 10 min and the pellet resuspended in cold phosphate-buffered saline (PBS) and again centrifuged in a two phase Percoll gradient (25%/50%). The central fraction containing LSEC and Kupffer cells (KC) was collected, washed with PBS, resuspended in LSEC media (RPMI 10% fetal bovine serum (FBS), 2 mM L-glutamine, 1% penicillin/streptomycin, 1% amphotericin B, 0.1mg/mL heparin and 0.05mg/mL endothelial cell growth supplement (ECGS)) and seeded in a non-coated plate for 30 min at 37ºC (5% CO_2). KC attached to the plate were discarded and the non-adherent LSEC were seeded in collagen-coated culture plates, incubated for 45 min (37ºC, 5% CO_2) and washed afterwards. The resulting cells were incubated at 37ºC in humid atmosphere with 5% CO_2 in LSEC media.

HSC isolation

Hepatic stellate cells (HSC) were isolated from CD-Veh (*n*=5), HFGFD-Veh (n=5), HFGFD-Sim (n=5) and HFGFD-Ato (n=5) rat livers as previously described (24). Briefly, livers were perfused with pronase, collagenase and DNase through the portal vein for 10min at a flow rate of 20 mL/min at 37ºC with Gey's Balanced Salt Solution (GBSS). Then, 30 min at a flow rate of 5 mL/min at 37ºC, excised and in vitro digested for 10 min at 37ºC, also with pronase, collagenase and Dnase. Resulting cells were filtered and centrifuged at 50 g for 5 min in order to eliminate the hepatocytes. The supernatant was then centrifuged at 800 g for 10 min and the pellet was resuspended in cold GBBS and centrifuged in Optiprep gradient (11%). The fraction containing HSC was collected, washed with GBSS, resuspended in HSC media (IMDM 10% FBS, 2 mM L-glutamine, 1% penicillin/streptomycin and 1% amphotericin B) and seeded in a non-coated plate at 37ºC (5% CO_2) overnight and washed afterwards.

Western blot analysis

Livers were perfused with saline for exsanguination and samples were directly frozen in liquid nitrogen, crushed to powder and homogenized in triton-lysis buffer (25 mM Tris/HCl pH 7.6, 137 mM NaCl, 2.7 mM KCl, 20 mM NaF, 10 mM $Na_4P_2O_7$, 10 nM okadaic acid, 2 mM Na_3VO_4, 2 µg/mL antipain, 2 µg/mL aprotinin, 2 µg/mL chymostatin, 2 µg/mL leupeptin, 2 µg/mL pepstatin A, 2 µg/mL trypsin inhibitor, 40 µg/mL phenylmethylsulfonylfluoride, and 10% v/v Triton X-100). HSC were washed with PBS and lysed using triton-lysis buffer. Homogenized livers and cell lysates were sonicated and centrifuged at 14000 g

at 4ºC for 10 min. Supernatant protein concentration was assessed by BCA ™ Protein Assay Kit (Thermo Fisher Scientific, Rockford, IL, USA).

Equal amounts of protein (60 µg of liver protein/lane and 40 µg of HSC protein/lane) were run on a 10% sodium dodecyl sulphate-polyacrylamide gel electrophoresis (SDS-PAGE). Proteins were blotted onto a polyvinylidene difluoride (PVDF) membrane (Thermo Fisher Scientific, Waltham, MA USA). Membranes were blocked in 5% phosphoblocker (Cell biolabs, San Diego, CA, USA) and incubated with primary antibodies: phosphorylated endothelial nitric oxide synthase (P-eNOS, Ser1177, 1/250), phosphorylated protein kinase B (P-Akt, 1/500), extracellular signal–regulated kinase (ERK1/2, 1/1000), P-ERK1/2 (Thr202/Tyr204, 1/1000) (Cell Signalling, Danvers, MA, USA), Kruppel-like factor 2 (KLF2, 1/200), P-moesin (1/200), early transcription factor (cFOS, 1/200) (Santa Cruz biotechnology, Dallas, TX, USA), alpha-smooth muscle actin (αSMA, 1/200) (Abcam, Cambridge, UK), endothelin receptor subtype B (ETRB, 1/200) and endothelin receptor subtype A (ETRA, 1/200) (Alomone Labs, Jerusalem, Israel). Glyceraldehyde-3-phosphate dehydrogenase (GAPDH, 1/5000, Ambion, Austin, TX, USA) was used as loading control. Thereafter, membranes were incubated with the corresponding secondary peroxidase-coupled antibody, developed using ECL kit (GE Healthcare; Little Chalfont, UK) and quantified by Image Studio Lite (Lincoln, NE, USA).

RT qPCR

RNA from different subtypes of LSEC, Kupffer Cells, HSC and hepatocytes was extracted using RNeasy Micro Kit (QIAGEN, Venlo, Nederland) and reverse-transcribed to complementary DNA (SuperScript™ VILO™ cDNA Synthesis Kit,

Thermo Fisher scientific, Waltham, MA, USA). Specific target amplification was accomplished using TaqMan PreAmp Master Mix (Applied Biosystems, Foster City, CA, USA) consisting of equal volumes of the different assays diluted with 1xTE buffer to a final concentration of 0.2x. Preamplification was performed at 95ºC for 10 min, followed by 14 cycles of 95ºC for 15 sec and 60ºC for 4 min, and a hold at 4ºC. Then, a dilution 1:10 of preamplified cDNA was added to Taqman universal PCR master mix plus the specific probe and loaded in 384-well plates (Thermo Fisher scientific, Waltham, MA, USA). Quantitative polymerase chain reaction (qPCR) was performed using 7900HT Fast Real-Time PCR system (Thermo Fisher scientific, Waltham, MA, USA) (initial stage at 50ºC for 2 min and 95ºC for 10 min, followed by 40 cycles of amplification of 95ºC for 15 s, 60ºC for 1 min). The relative gene expression was normalized to glyceraldehyde-3-phosphate dehydrogenase (GAPDH). Data was analyzed using the Relative Quantification qPCR Application in Thermofisher Cloud.

The following specific probes were used: Kruppel-like factor 2 (KLF2); eNOS; platelet endothelial cell adhesion molecule (CD31, PECAM-1); endothelin-1 (ET-1); Stabilin1 (STAB1); lymphatic vessel endothelial hyaluronan receptor 1 (LYVE1); Fc gamma receptor IIb (CD32b, Fcgr2b); F4/80 (Emr1); Cyp3A2; Desmin and GAPDH.

LEGENDS

Supplementary Figure 1. Study design diagram. Study design and assignation of animals for the different experiments. The study design comprised three cohorts of rats. **(A)** One cohort was dedicated to biochemical, histological and hemodynamic study. Animals with muscular toxicity were discarded for further analysis. The HOMA index and whole liver sample analysis were performed in animals from these groups. **(B)** The second cohort was used for the isolation of liver sinusoidal endothelial cells (LSEC) and the third one **(C)** for the hepatic stellate cells (HSC) isolation. The n values are for biological replicates.

Supplementary Figure 2. Bodyweight changes and insulin resistance. (A) Body weight gain was assessed at the end of week 10 in control diet (CD-Veh, n=15), and high-fat glucose-fructose diet rats treated with vehicle (HFGFD-Veh, n=15), simvastatin (HFGFD-Sim, n=12) or atorvastatin (HFGFD-Ato, n=14), compared to CD-Veh. **(**B) Homeostasis model of insulin resistance (HOMA-IR) in control diet (CD-Veh, n=7), and high-fat glucose-fructose diet rats treated with vehicle (HFGFD-Veh, n=7), simvastatin (HFGFD-Sim, n=8) or atorvastatin (HFGFD-Ato, n=10). Values are expressed as mean ± SEM; *p<0.05, **p<0.01, ***p<0,001 compared to CD-Veh; #p<0.05 compared to HFGFD-Veh.

Supplementary Figure 3. Western blot analysis of intrahepatic endothelial dysfunction markers. (A) Bar diagrams show protein quantification of KLF2, P-AKT and P-eNOS in control diet (CD-Veh, n=7), and high-fat glucose-fructose diet rats treated with vehicle (HFGFD-Veh, n=7), simvastatin (HFGFD-Sim, n=6) or atorvastatin (HFGFD-Ato, n=7). Protein levels are normalized to HFGFD-Veh

group and expressed as mean ± SEM. (B) Corresponding Western blots are shown below. GAPDH was used as loading control.

Supplementary Figure 4. **Gene expression of cell type specific markers in CD32b⁻ LSEC.** Relative quantitation of mRNA expression of A) F4/80, B) Cyp3A2, and C) Desmin in CD32b⁻/CD11b/c⁻ LSEC isolated from CD-Veh (n=4) and HFGFD rats after vehicle (HFGFD-Veh, n=4), simvastatin (HFGFD-Sim, n=4) or atorvastatin (HFGFD-Ato, n=4) treatment. Results are expressed as fold change normalized to the expression of specific cell markers in each reference cell type. GAPDH was used as endogenous control. mRNA levels are expressed as mean ± SEM.

Supplementary Figure 5. Western blot for the analysis of endothelin pathway markers in HSC. Full-length gels for the analysis of protein expression of P-ERK1/2, ERK1/2, P-moesin, cFOS, αSMA, ETRA, ETRB and GAPDH in HSC from control diet (CD-Veh, n=5), and high-fat glucose-fructose diet rats treated with vehicle (HFGFD-Veh, n=5), simvastatin (HFGFD-Sim, n=5) or atorvastatin (HFGFD-Ato, n=5). Note that some animals from HFGFD-Veh group have been repeated between gels in order to normalize intensities.

Supplementary Table 1. NASH Clinical Research Network Scoring System Definitions.

Item	Definition	Score
**Steatosis**	Low- to medium-power evaluation of parenchymal involvement by steatosis	
	<5%	0
	5%-33%	1
	33%-66%	2
	>66%	3
**Lobular inflammation**	Overall assessment of all inflammatory foci	
	No foci	0
	<2 foci per 200x field	1
	2-4 foci per 200x field	2
	>4 foci per 200x field	3
**Hepatocellular ballooning**	None	1
	Few balloon cells	2
	Many cells/prominent ballooning	3

Supplementary Table 2. Biochemical parameters.

	CD Vehicle	HFGFD Vehicle	HFGFD Simvastatin	HFGFD Atorvastatin
**Glucose (mg/dL)**	209.87±20.14	233.33±33.53	195±23.35	219.29±20.12
**Insulin (ng/ml)**	11.668±2.57	24.228±2.34###	16.377±1.91**	15.565±1.12***
**Creatinine (mg/dL)**	0.75±0.02	0.79±0.04	0.92±0.08	0.80±0.05
**Bilirrubin (mg/dL)**	0.08±0.01	0.11±0.01	0.09±0.01	0.11±0.01
**AST (UI/L)**	182.75±33.39	146.83±14.27	194.6±47.83	109.00±7.61
**ALT (UI/L)**	40.93±2.61	49.47±2.72#	48.27±5.02	38.07±2**
**AP (UI/L)**	134.47±7.15	154.13±11.70	144.67±9.32	132.50±7.22
**CK (UI/L)**	2634.57±630.59	2265±528.67	2243.75±497.47	1220.71±164.71
**Cholesterol (mg/dL)**	81.4±3.55	79.8±4.03	67.63±3.98	74.43±3.45
**HDL (mg/dL)**	47.67±2.27	42.6±2.04	45.67±4.2	47.14±2.19
**LDL (mg/dL)**	28.07±1.52	28.67±2.93	23.5±4.66	21.79±1.95
**Triglycerides (mg/dL)**	29.07±1.88	42.33±4.35##	31.75±2.79*	28±1.83***
**Albumin (g/dL)**	2.51±0.06	2.82±0.06###	2.91±0.08###	2.74±0.04##

Values are expressed as mean ± SEM in CD-Vehicle (n=15). HFGFD-Vehicle (n=15). HFGFD-Simvastatin (n=12) and HFGFD-Atorvastatin (n=14). AST, aspartate aminotransferase; ALT, alanine aminotransferase; AP, alkaline phosphatase; CK, creatine kinase. *p<0.05, **p<0.01, ***p<0.001 compared with HFGFD-vehicle; #p<0.05, ##p<0.01, ###p<0.001 compared with CD-vehicle.

Supplementary Table 3. Adverse events after two weeks treatment with simvastatin (10mg·Kg^{-1}·day^{-1}) or atorvastatin (10mg·Kg^{-1}·day^{-1}).

	n	Hepatic toxicity rate (%)	Muscular toxicity rate (%)
**CD-Vehicle**	**15**	**0**	**0**
**HFGFD-Vehicle**	**15**	**0**	**0**
**HFGFD-Simvastatin**	**13**	**0**	**7.69**
**HFGFD-Atorvastatin**	**16**	**0**	**12.50**

N, number of rats; hepatic toxicity rate, % of animals with serum ALT levels > 150 IU/L; muscular toxicity rate, % of animals with serum CK levels > 8400 IU/L.

Supplementary Figure 1

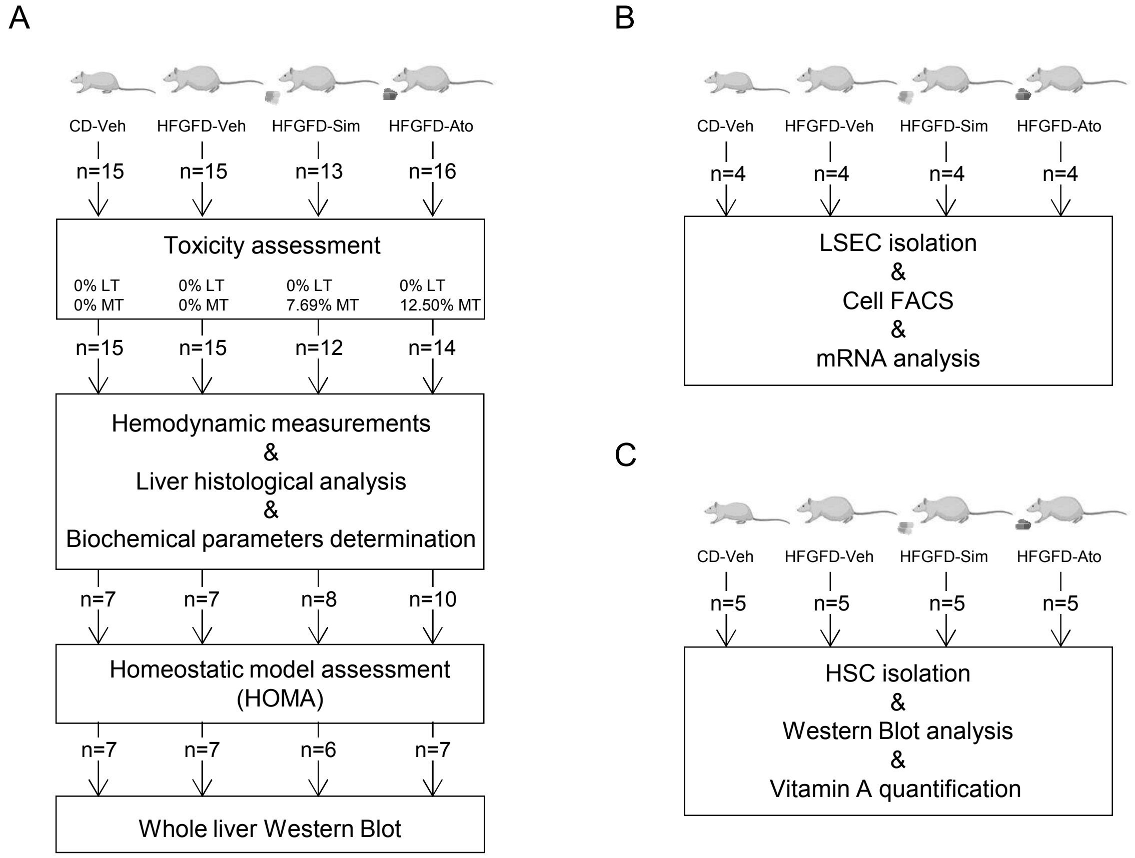

Supplementary Figure 2

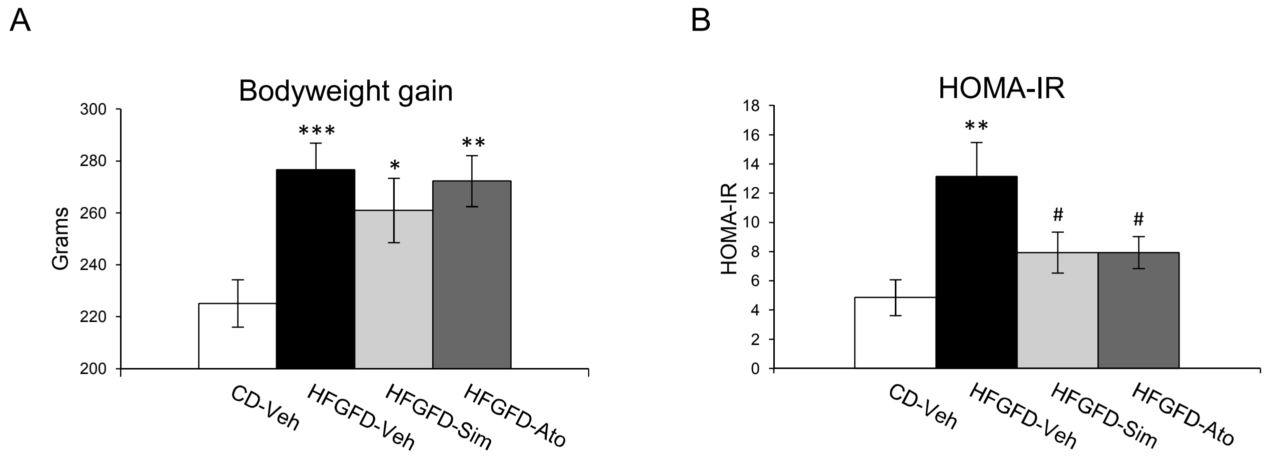

Supplementary Figure 3

A

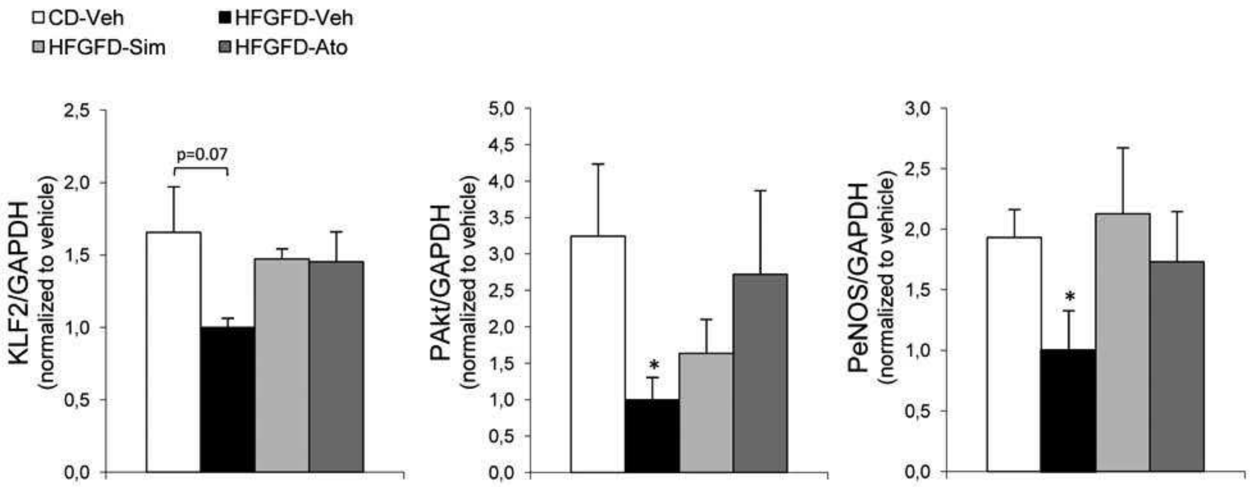

B

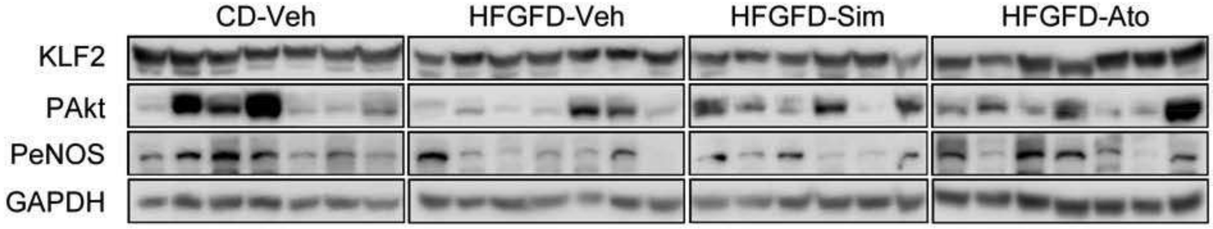

Supplementary Figure 4

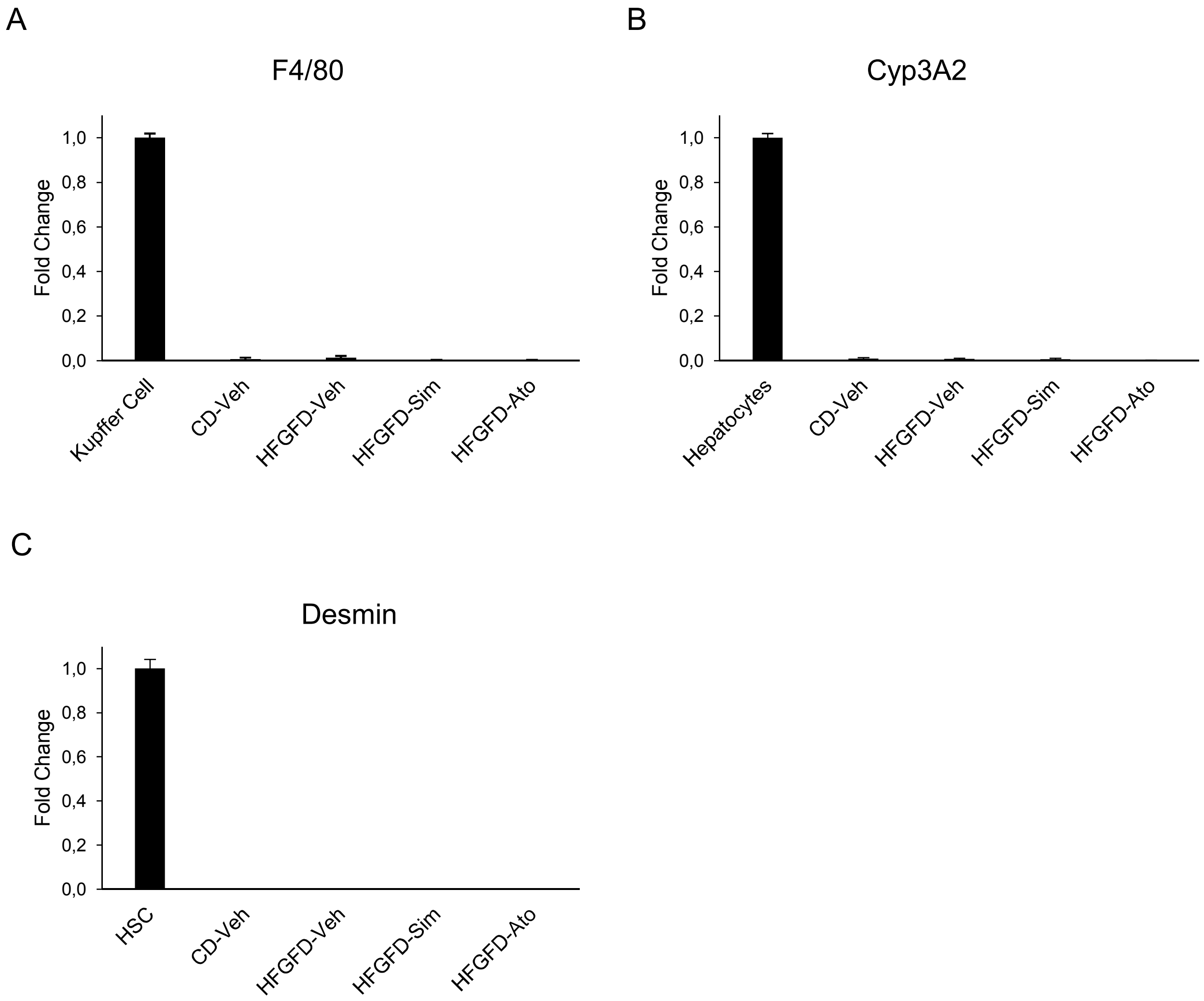

Supplementary Figure 5

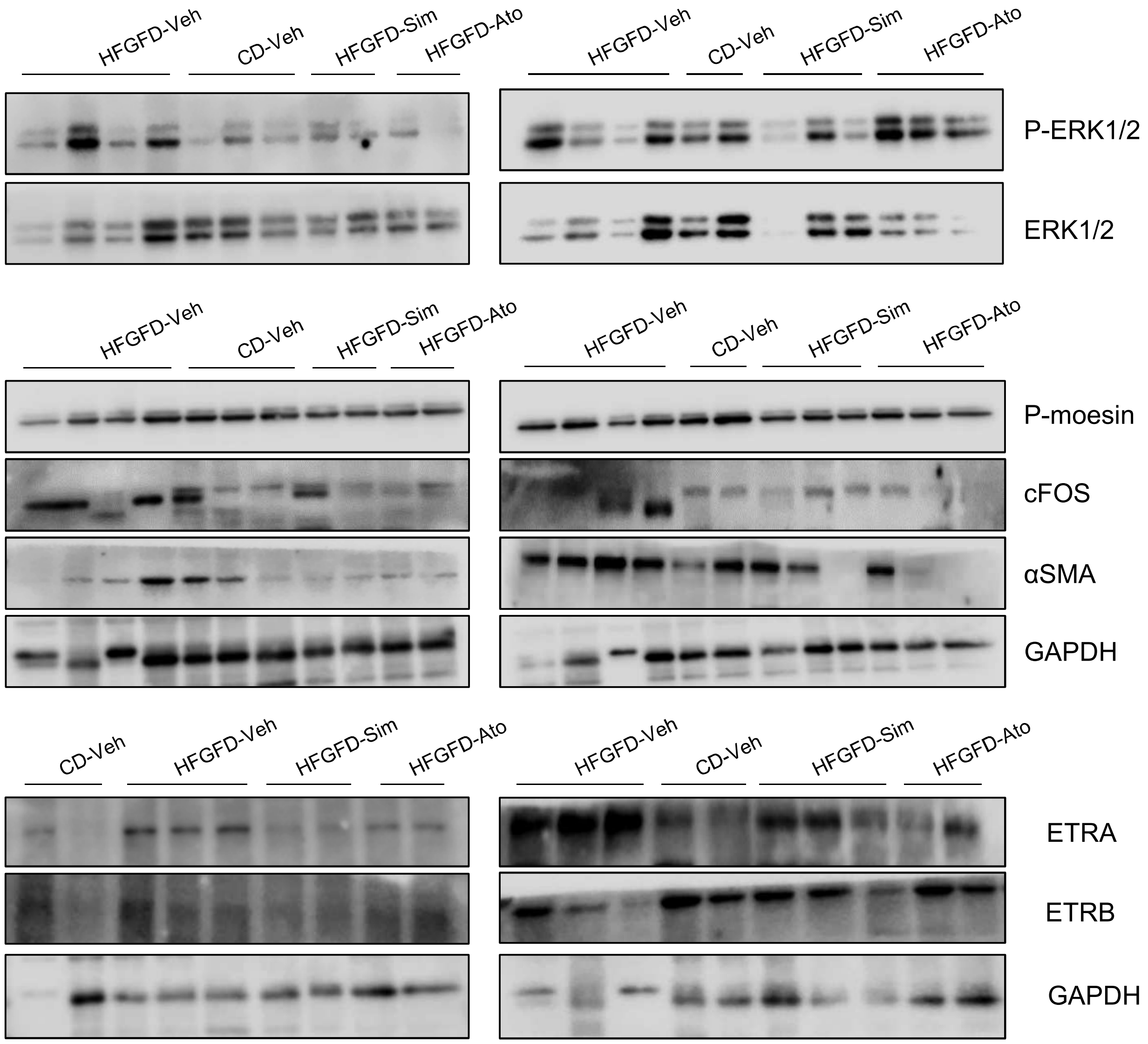

Supplement: Supplementary file 1 — Supplementary Information [file 41598_2019_56366_MOESM1_ESM.pdf]
